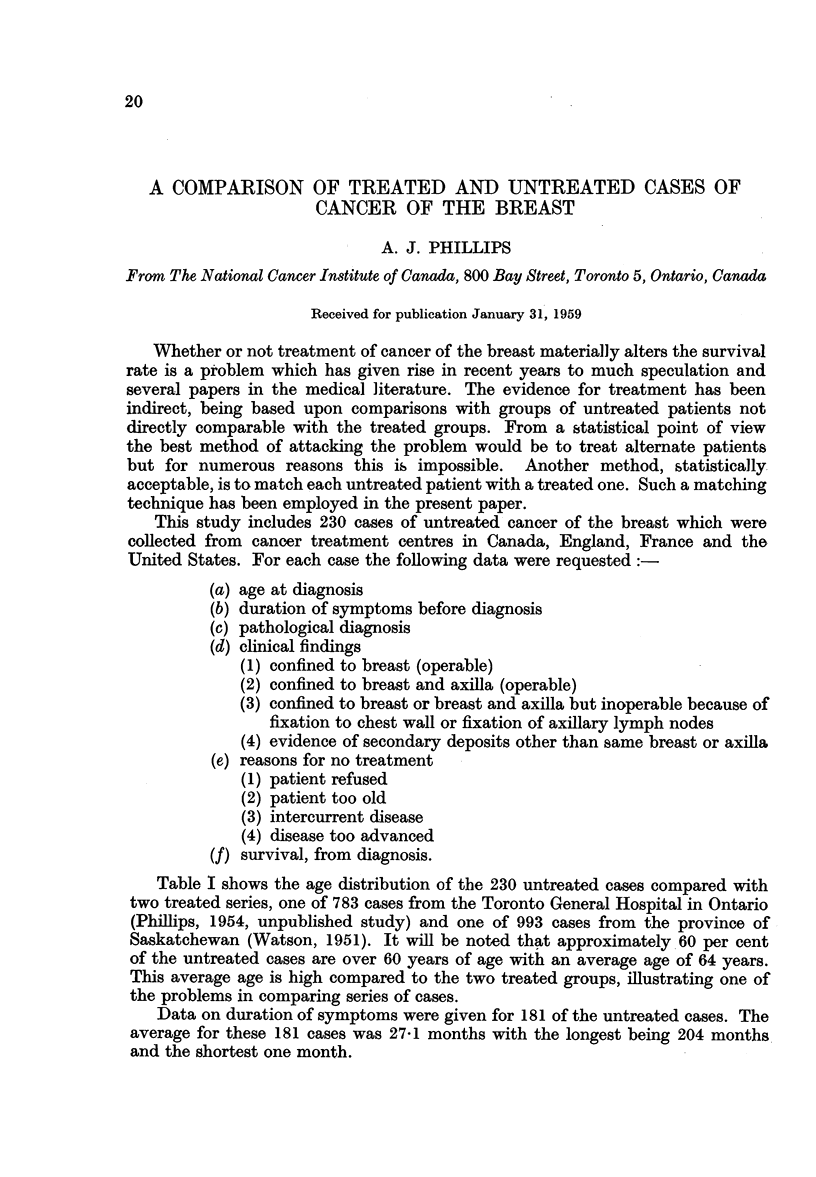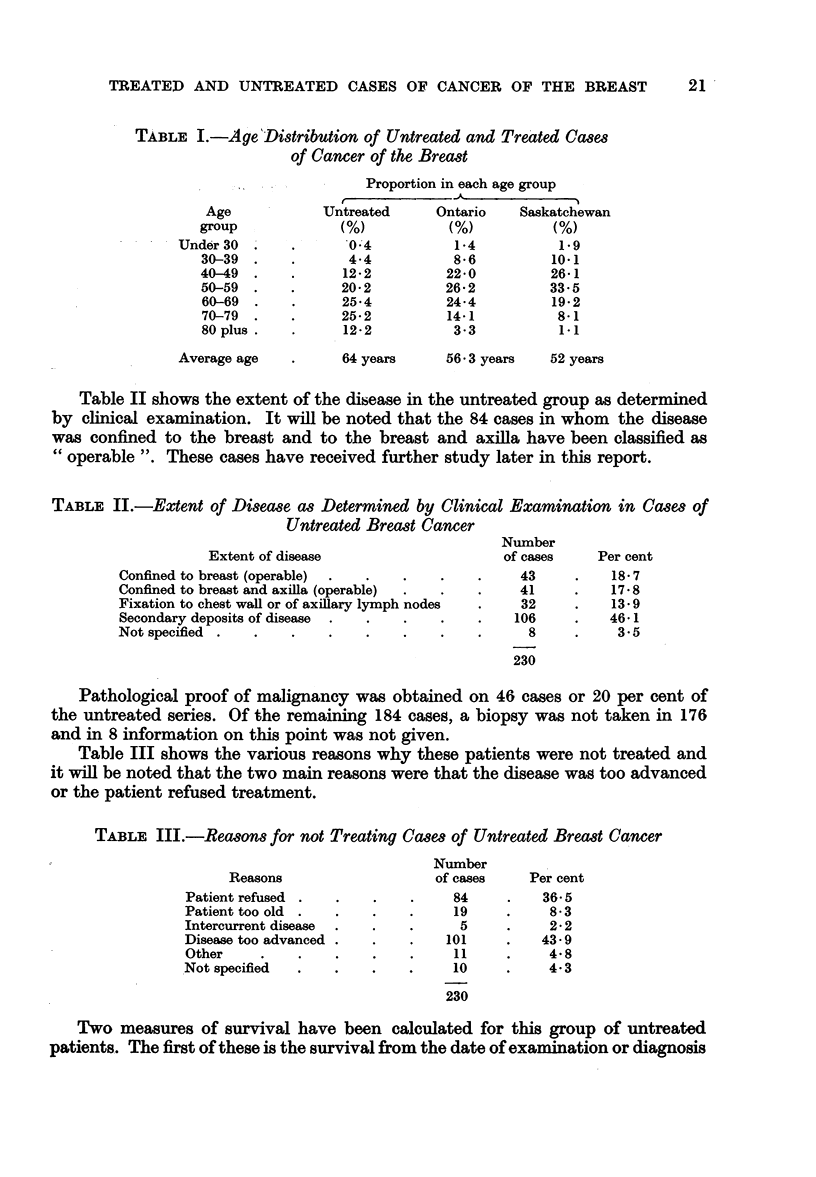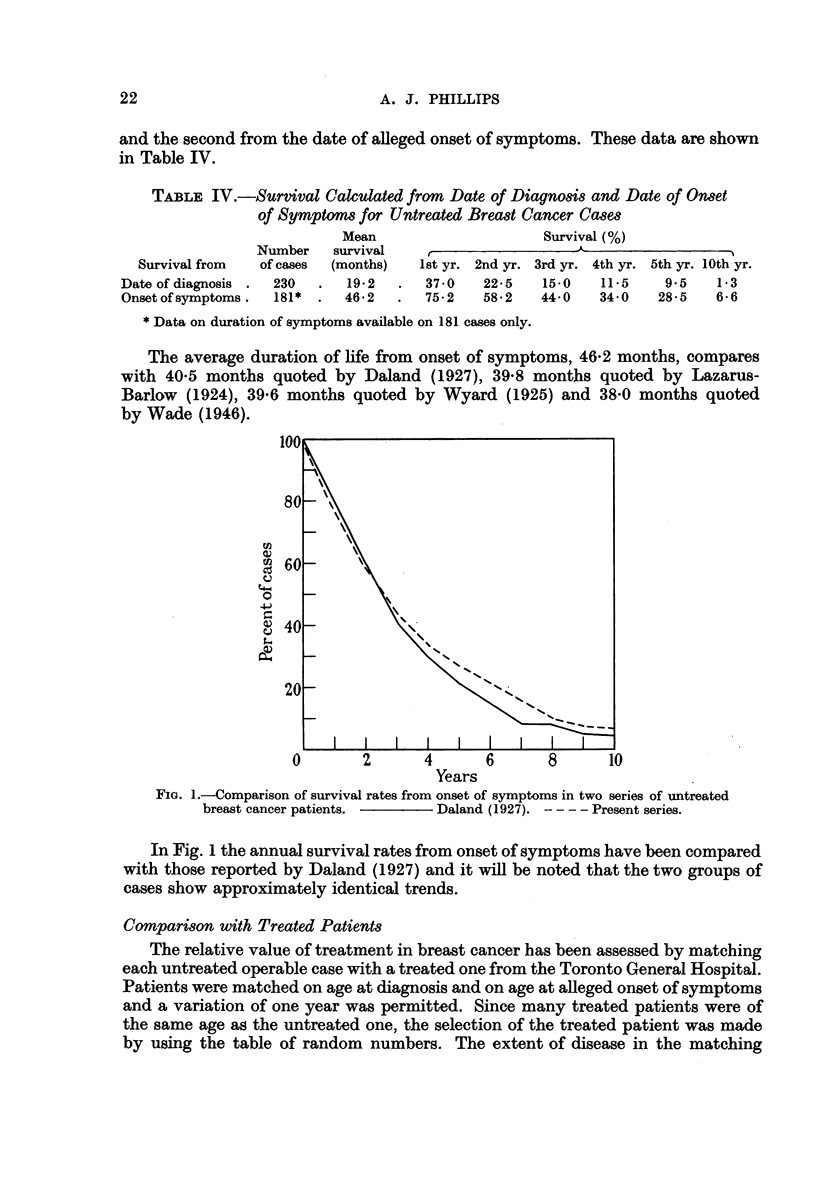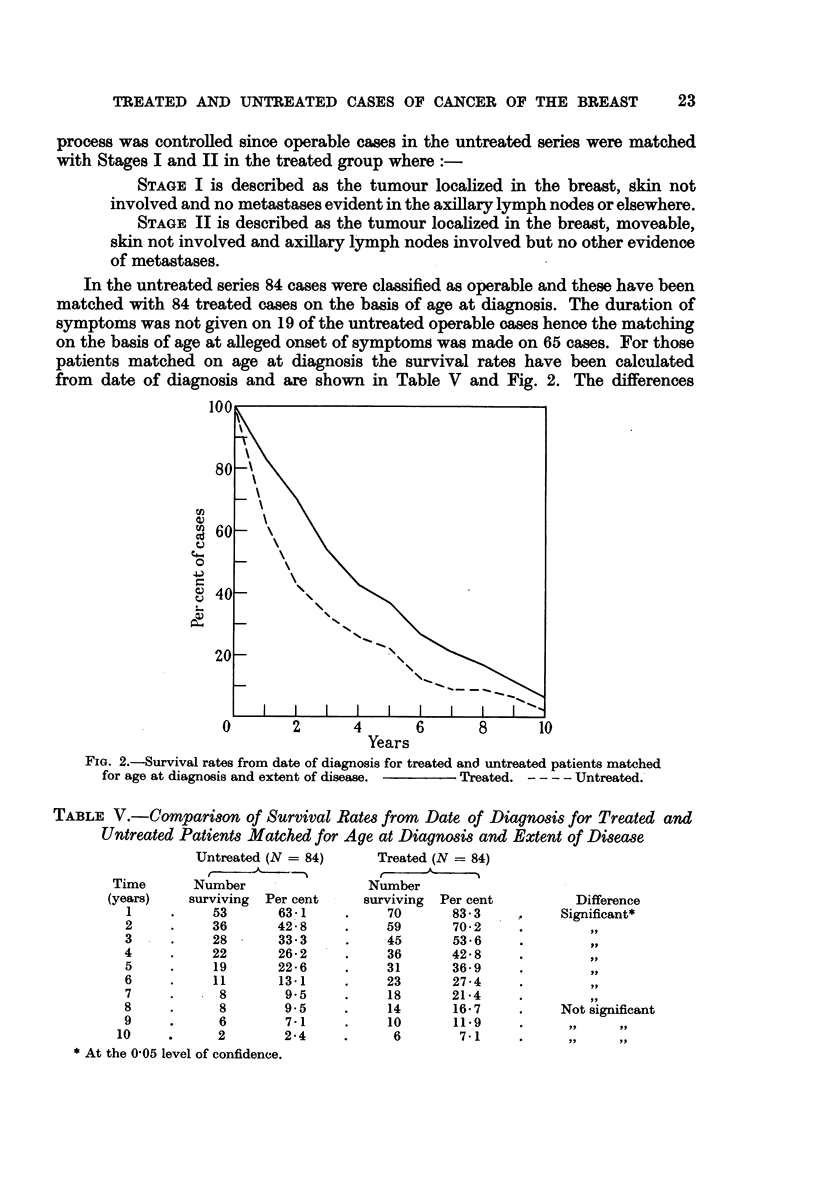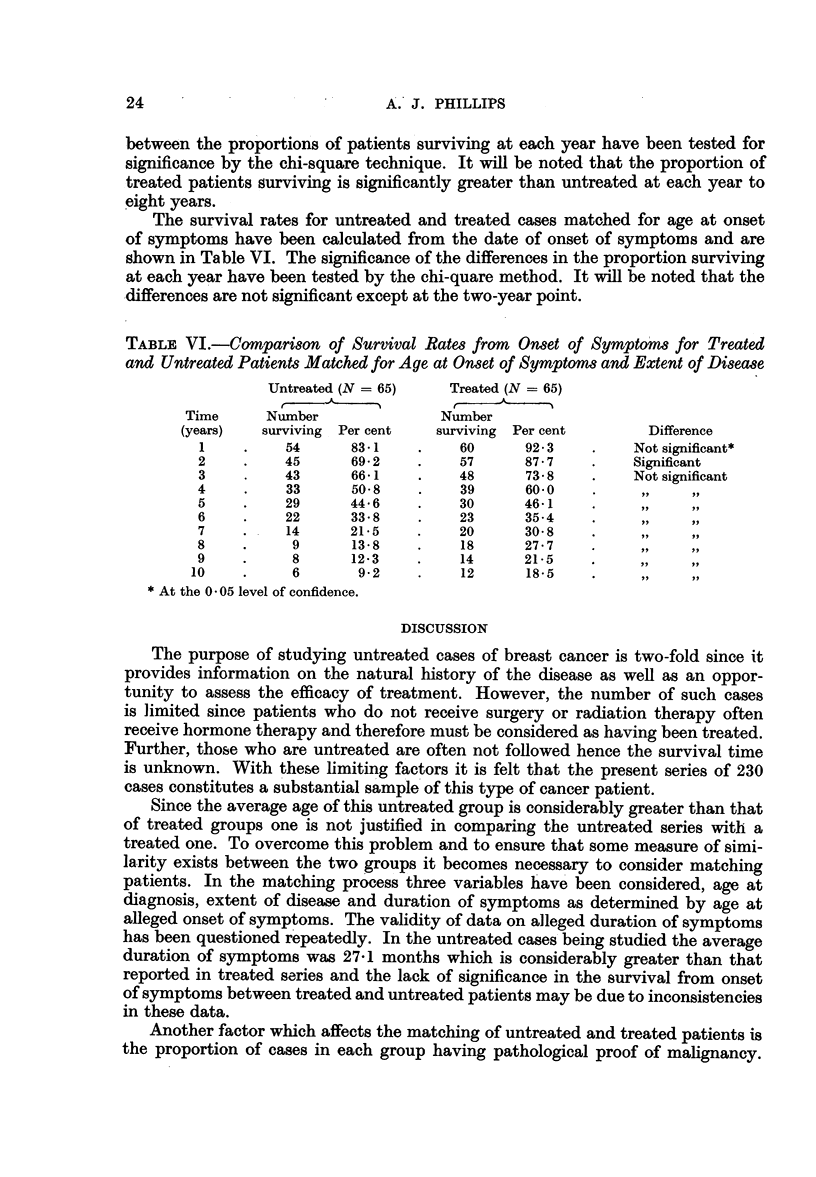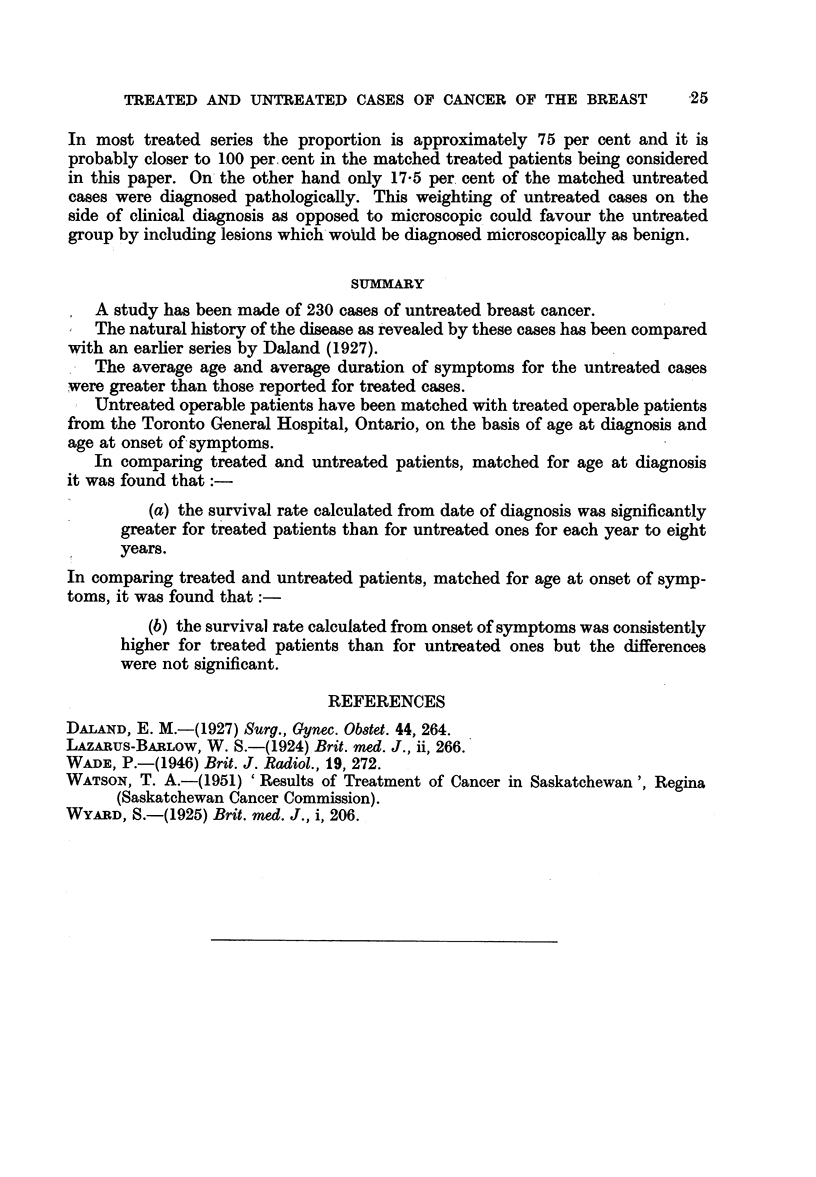# A Comparison of Treated and Untreated Cases of Cancer of the Breast

**DOI:** 10.1038/bjc.1959.3

**Published:** 1959-03

**Authors:** A. J. Phillips


### A COMPARISON OF TREATED AND UNTREATED CASES OF CANCER OF THE BREAST

A. J. PHILLIPS

*From The National Cancer Institute of Canada,* 800 *Bay Street, Toronto* 5, *Ontario, Canada*

Received for publication January 31, 1959

Whether or not treatment of cancer of the breast materially alters the survival rate is a problem which has given rise in recent years to much speculation and several papers in the medical literature. The evidence for treatment has been indirect, being based upon comparisons with groups of untreated patients not directly comparable with the treated groups. From a statistical point of view the best method of attacking the problem would be to treat alternate patients but for numerous reasons this is impossible. Another method, statistically acceptable, is to match each untreated patient with a treated one. Such a matching technique has been employed in the present paper.

This study includes 230 cases of untreated cancer of the breast which were collected from cancer treatment centres in Canada, England, France and the United States. For each case the following data were requested :—

(*a*) age at diagnosis
(*b*) duration of symptoms before diagnosis
(*c*) pathological diagnosis
(*d*) clinical findings
  (1) confined to breast (operable)
  (2) confined to breast and axilla (operable)
  (3) confined to breast or breast and axilla but inoperable because of fixation to chest wall or fixation of axillary lymph nodes
  (4) evidence of secondary deposits other than same breast or axilla
(*e*) reasons for no treatment
  (1) patient refused
  (2) patient too old
  (3) intercurrent disease
  (4) disease too advanced
(*f*) survival, from diagnosis.

Table I shows the age distribution of the 230 untreated cases compared with two treated series, one of 783 cases from the Toronto General Hospital in Ontario (Phillips, 1954, unpublished study) and one of 993 cases from the province of Saskatchewan (Watson, 1951). It will be noted that approximately 60 per cent of the untreated cases are over 60 years of age with an average age of 64 years. This average age is high compared to the two treated groups, illustrating one of the problems in comparing series of cases.

Data on duration of symptoms were given for 181 of the untreated cases. The average for these 181 cases was 27·1 months with the longest being 204 months and the shortest one month.

TABLE I.—*Age Distribution of Untreated and Treated Cases of Cancer of the Breast*

| Age group | Proportion in each age group: Untreated (%) | Ontario (%) | Saskatchewan (%) |
|---|---|---|---|
| Under 30 | 0·4 | 1·4 | 1·9 |
| 30–39 | 4·4 | 8·6 | 10·1 |
| 40–49 | 12·2 | 22·0 | 26·1 |
| 50–59 | 20·2 | 26·2 | 33·5 |
| 60–69 | 25·4 | 24·4 | 19·2 |
| 70–79 | 25·2 | 14·1 | 8·1 |
| 80 plus | 12·2 | 3·3 | 1·1 |
| Average age | 64 years | 56·3 years | 52 years |

Table II shows the extent of the disease in the untreated group as determined by clinical examination. It will be noted that the 84 cases in whom the disease was confined to the breast and to the breast and axilla have been classified as " operable ". These cases have received further study later in this report.

TABLE II.—*Extent of Disease as Determined by Clinical Examination in Cases of Untreated Breast Cancer*

| Extent of disease | Number of cases | Per cent |
|---|---|---|
| Confined to breast (operable) | 43 | 18·7 |
| Confined to breast and axilla (operable) | 41 | 17·8 |
| Fixation to chest wall or of axillary lymph nodes | 32 | 13·9 |
| Secondary deposits of disease | 106 | 46·1 |
| Not specified | 8 | 3·5 |
| | 230 | |

Pathological proof of malignancy was obtained on 46 cases or 20 per cent of the untreated series. Of the remaining 184 cases, a biopsy was not taken in 176 and in 8 information on this point was not given.

Table III shows the various reasons why these patients were not treated and it will be noted that the two main reasons were that the disease was too advanced or the patient refused treatment.

TABLE III.—*Reasons for not Treating Cases of Untreated Breast Cancer*

| Reasons | Number of cases | Per cent |
|---|---|---|
| Patient refused | 84 | 36·5 |
| Patient too old | 19 | 8·3 |
| Intercurrent disease | 5 | 2·2 |
| Disease too advanced | 101 | 43·9 |
| Other | 11 | 4·8 |
| Not specified | 10 | 4·3 |
| | 230 | |

Two measures of survival have been calculated for this group of untreated patients. The first of these is the survival from the date of examination or diagnosis and the second from the date of alleged onset of symptoms. These data are shown in Table IV.

TABLE IV.—*Survival Calculated from Date of Diagnosis and Date of Onset of Symptoms for Untreated Breast Cancer Cases*

| Survival from | Number of cases | Mean survival (months) | Survival (%) 1st yr. | 2nd yr. | 3rd yr. | 4th yr. | 5th yr. | 10th yr. |
|---|---|---|---|---|---|---|---|---|
| Date of diagnosis | 230 | 19·2 | 37·0 | 22·5 | 15·0 | 11·5 | 9·5 | 1·3 |
| Onset of symptoms | 181* | 46·2 | 75·2 | 58·2 | 44·0 | 34·0 | 28·5 | 6·6 |

* Data on duration of symptoms available on 181 cases only.

The average duration of life from onset of symptoms, 46·2 months, compares with 40·5 months quoted by Daland (1927), 39·8 months quoted by Lazarus-Barlow (1924), 39·6 months quoted by Wyard (1925) and 38·0 months quoted by Wade (1946).

FIG. 1.—Comparison of survival rates from onset of symptoms in two series of untreated breast cancer patients. ———— Daland (1927). – – – – Present series.

In Fig. 1 the annual survival rates from onset of symptoms have been compared with those reported by Daland (1927) and it will be noted that the two groups of cases show approximately identical trends.

*Comparison with Treated Patients*

The relative value of treatment in breast cancer has been assessed by matching each untreated operable case with a treated one from the Toronto General Hospital. Patients were matched on age at diagnosis and on age at alleged onset of symptoms and a variation of one year was permitted. Since many treated patients were of the same age as the untreated one, the selection of the treated patient was made by using the table of random numbers. The extent of disease in the matching process was controlled since operable cases in the untreated series were matched with Stages I and II in the treated group where :—

STAGE I is described as the tumour localized in the breast, skin not involved and no metastases evident in the axillary lymph nodes or elsewhere.

STAGE II is described as the tumour localized in the breast, moveable, skin not involved and axillary lymph nodes involved but no other evidence of metastases.

In the untreated series 84 cases were classified as operable and these have been matched with 84 treated cases on the basis of age at diagnosis. The duration of symptoms was not given on 19 of the untreated operable cases hence the matching on the basis of age at alleged onset of symptoms was made on 65 cases. For those patients matched on age at diagnosis the survival rates have been calculated from date of diagnosis and are shown in Table V and Fig. 2. The differences

FIG. 2.—Survival rates from date of diagnosis for treated and untreated patients matched for age at diagnosis and extent of disease. ——— Treated. - - - - Untreated.

TABLE V.—*Comparison of Survival Rates from Date of Diagnosis for Treated and Untreated Patients Matched for Age at Diagnosis and Extent of Disease*

| Time (years) | Untreated (N = 84) Number surviving | Untreated (N = 84) Per cent | Treated (N = 84) Number surviving | Treated (N = 84) Per cent | Difference |
|---|---|---|---|---|---|
| 1 | 53 | 63·1 | 70 | 83·3 | Significant* |
| 2 | 36 | 42·8 | 59 | 70·2 | ” |
| 3 | 28 | 33·3 | 45 | 53·6 | ” |
| 4 | 22 | 26·2 | 36 | 42·8 | ” |
| 5 | 19 | 22·6 | 31 | 36·9 | ” |
| 6 | 11 | 13·1 | 23 | 27·4 | ” |
| 7 | 8 | 9·5 | 18 | 21·4 | ” |
| 8 | 8 | 9·5 | 14 | 16·7 | Not significant |
| 9 | 6 | 7·1 | 10 | 11·9 | ” ” |
| 10 | 2 | 2·4 | 6 | 7·1 | ” ” |

* At the 0·05 level of confidence.

between the proportions of patients surviving at each year have been tested for significance by the chi-square technique. It will be noted that the proportion of treated patients surviving is significantly greater than untreated at each year to eight years.

The survival rates for untreated and treated cases matched for age at onset of symptoms have been calculated from the date of onset of symptoms and are shown in Table VI. The significance of the differences in the proportion surviving at each year have been tested by the chi-quare method. It will be noted that the differences are not significant except at the two-year point.

TABLE VI.—*Comparison of Survival Rates from Onset of Symptoms for Treated and Untreated Patients Matched for Age at Onset of Symptoms and Extent of Disease*

| | Untreated ($N$ = 65) | | Treated ($N$ = 65) | | |
|---|---|---|---|---|---|
| Time (years) | Number surviving | Per cent | Number surviving | Per cent | Difference |
| 1 | 54 | 83·1 | 60 | 92·3 | Not significant* |
| 2 | 45 | 69·2 | 57 | 87·7 | Significant |
| 3 | 43 | 66·1 | 48 | 73·8 | Not significant |
| 4 | 33 | 50·8 | 39 | 60·0 | ,, ,, |
| 5 | 29 | 44·6 | 30 | 46·1 | ,, ,, |
| 6 | 22 | 33·8 | 23 | 35·4 | ,, ,, |
| 7 | 14 | 21·5 | 20 | 30·8 | ,, ,, |
| 8 | 9 | 13·8 | 18 | 27·7 | ,, ,, |
| 9 | 8 | 12·3 | 14 | 21·5 | ,, ,, |
| 10 | 6 | 9·2 | 12 | 18·5 | ,, ,, |

* At the 0·05 level of confidence.

### DISCUSSION

The purpose of studying untreated cases of breast cancer is two-fold since it provides information on the natural history of the disease as well as an opportunity to assess the efficacy of treatment. However, the number of such cases is limited since patients who do not receive surgery or radiation therapy often receive hormone therapy and therefore must be considered as having been treated. Further, those who are untreated are often not followed hence the survival time is unknown. With these limiting factors it is felt that the present series of 230 cases constitutes a substantial sample of this type of cancer patient.

Since the average age of this untreated group is considerably greater than that of treated groups one is not justified in comparing the untreated series with a treated one. To overcome this problem and to ensure that some measure of similarity exists between the two groups it becomes necessary to consider matching patients. In the matching process three variables have been considered, age at diagnosis, extent of disease and duration of symptoms as determined by age at alleged onset of symptoms. The validity of data on alleged duration of symptoms has been questioned repeatedly. In the untreated cases being studied the average duration of symptoms was 27·1 months which is considerably greater than that reported in treated series and the lack of significance in the survival from onset of symptoms between treated and untreated patients may be due to inconsistencies in these data.

Another factor which affects the matching of untreated and treated patients is the proportion of cases in each group having pathological proof of malignancy.

In most treated series the proportion is approximately 75 per cent and it is probably closer to 100 per cent in the matched treated patients being considered in this paper. On the other hand only 17·5 per cent of the matched untreated cases were diagnosed pathologically. This weighting of untreated cases on the side of clinical diagnosis as opposed to microscopic could favour the untreated group by including lesions which would be diagnosed microscopically as benign.

SUMMARY

A study has been made of 230 cases of untreated breast cancer.

The natural history of the disease as revealed by these cases has been compared with an earlier series by Daland (1927).

The average age and average duration of symptoms for the untreated cases were greater than those reported for treated cases.

Untreated operable patients have been matched with treated operable patients from the Toronto General Hospital, Ontario, on the basis of age at diagnosis and age at onset of symptoms.

In comparing treated and untreated patients, matched for age at diagnosis it was found that :—

(*a*) the survival rate calculated from date of diagnosis was significantly greater for treated patients than for untreated ones for each year to eight years.

In comparing treated and untreated patients, matched for age at onset of symptoms, it was found that :—

(*b*) the survival rate calculated from onset of symptoms was consistently higher for treated patients than for untreated ones but the differences were not significant.

### REFERENCES

DALAND, E. M.—(1927) *Surg., Gynec. Obstet.* **44**, 264.
LAZARUS-BARLOW, W. S.—(1924) *Brit. med. J.*, ii, 266.
WADE, P.—(1946) *Brit. J. Radiol.*, **19**, 272.
WATSON, T. A.—(1951) 'Results of Treatment of Cancer in Saskatchewan', Regina (Saskatchewan Cancer Commission).
WYARD, S.—(1925) *Brit. med. J.*, i, 206.